# Personal

**Published:** 1912-04

**Authors:** 


### PERSONAL

---

READERS are requested to send items for this section.

---

DR. LAWRENCE HENDEE of Buffalo, returned from Atlantic City, March 7.

---

DR. CHARLES A. BRADSHAW of Syracuse spent several days in Buffalo last month.

---

DR. EDWARD W. ROOS of Buffalo has returned from Europe and will locate in Corning.

---

DR. EVA G. FOWLER of Buffalo has been appointed resident physician at the State Training School for Girls at Hudson.

DR. EDWARD H. HUME, physician in charge of the Yale Hospital at Changsha, China, gave an illustrated lecture on the Significance of the Chinese Revolution, at the University Club of Buffalo, March 2, 1912.

DR. BRADFORD A. RICHARDS of Rochester, has removed his office from 6 Gibbs Street to 64 Clinton Ave. S. having become associated with Dr. John O. Roe.

DR. JESSE FLEET SAMMIS is removing from Rochester to New York city.

DR. S. CASE JONES, Rochester, has removed his office to 116 Barrington St.

DR. JOHN M. SWAN spoke before the Rochester Academy of Science upon the biology of the malarial parasites.

DR. FREDERICK W. ZIMMER of Rochester is on a trip to Panama.

DR. LEGRAND ALLEN WALKER, Rochester, N. Y., has returned to his home after an operation for appendicitis.

DR. J. W. KENNEDY of Philadelphia, gave a paper before the Rochester Pathological Society, March 21, on "Drainage of the Suppurative Lesions of the Abdomen."

DR. JOHN R. WILLIAMS and Dr. Joseph Roby discussed problems of milk and bovine tuberculosis before the Monroe County Medical Society on March 19.

DR. HENRY H. COVELL of Rochester, has left for a trip to New Orleans and other Southern points.

THE Rochester Chamber of Commerce has appointed the following Public Health Committee: Dr. Edward W. Mulligan, Chairman, Dr. J. R. Culkin, Dr. C. R. Barber, Dr. D. B. Jewett, Dr. L. L. Button and Dr. R. R. Fitch.

DR. E. R. LINKLATER of Buffalo, is seriously sick with scarlet fever.

DR. O. S. MCKEE of Buffalo, has returned from Atlantic City.

In noting the removal of Dr. John M. Swan from the Glen Springs to take up private practice in Rochester, his street number was given incorrectly. It should be 457 Park Ave.

---

Dr. Burt C. Johnson of Buffalo has returned from a fortnight's southern trip.

---

Dr. Milton P. Messinger of Oakfield, has recently purchased an apartment house.

---

Dr. Fred S. Hoffmann of Buffalo is improving some property in the vicinity of his residence.

---

Dr. Mary T. Greene of Castile has returned from Bermuda. Dr. Helen DeWitt Justin of Castile is spending several weeks in New York.

---

Dr. Sarah L. Cushing of Lockport, a well kown philanthropist was nearly asphyxiated by escaping gas, March 23. In spite of her 96 years, she is expected to recover.

---

Dr. Greenfel, the Labrador physician and missionary will address the Twentieth Century Club of Buffalo, April 6.

